# Mothers of children with down syndrome: A qualitative study of experiences of breastfeeding and breastfeeding support

**DOI:** 10.1111/scs.13088

**Published:** 2022-05-18

**Authors:** Lisbeth Jönsson, Christina Olsson Tyby, Sara Hullfors, Pia Lundqvist

**Affiliations:** ^1^ Department of Health Sciences Faculty of Medicine Lund University Lund Sweden

**Keywords:** breastfeeding, breastfeeding support, down syndrome, experiences, mothers

## Abstract

**Background:**

Children with down syndrome (DS) are breastfed to a lesser extent than infants in general, despite research showing that it is possible for these children to breastfeed successfully.

**Aim:**

The aim was to describe how mothers of children with DS experienced breastfeeding and breastfeeding support from healthcare professionals.

**Method:**

A qualitative study with an inductive approach. Individual interviews were performed with seven mothers from southern Sweden. The interviews were analysed using qualitative content analysis.

**Result:**

The mothers felt that the support varied, as some healthcare professionals were supportive, while others had preconceptions regarding breastfeeding and DS. They also experienced that the guidelines could be an obstacle in the encounter with healthcare professionals thereby affecting the possibility to establish breastfeeding. Information and support were important to the mothers, and when insufficient, they turned to the internet for help.

**Conclusions:**

Mothers felt that healthcare professionals were bound to ward routines and guidelines, which could be contrary to their own and the family's wishes. They were also sensitive to the attitudes of healthcare professionals, which can affect their own state of mind. Healthcare professionals' preconceptions regarding breastfeeding and DS have not changed, despite research showing that infants with DS can breastfeed successfully. Increased awareness of the possibility to breastfeed an infant with DS is needed to provide better support to mothers.

## INTRODUCTION

According to the World Health Organisation (WHO), the estimated incidence of children with down syndrome (DS) worldwide is between 1 in 1000 and 1 in 1100 live births. This means that approximately 3000–5000 children are born with this chromosomal disorder each year [1]. In Sweden, the corresponding figure is approximately 1.5/1000 infants. The number of children born with DS has decreased in Sweden [2].

Some of the characteristics associated with DS are large tongue muscle, lowered muscle tone, high palate and weakened suction ability. Consequently, feeding difficulties are common among infants with DS and initiating breastfeeding can be problematic [3]. According to the WHO, exclusive breastfeeding is recommended during an infant's first 6 months. Sweden follows these recommendations by supporting and encouraging breastfeeding [4]. Special guidelines on how healthcare professionals can support and promote breastfeeding have been developed in association with UNICEF [5]. These recommendations apply to all countries and populations irrespective of developmental level and economic status.

Breastfeeding has benefits. The content of breastmilk is individually composed to contain all the nutrition that the newborn infant needs during its first 6 months [3]. Breastfeeding protects the infant not only against various infections [6] but also against sudden infant death syndrome (SIDS) [7, 8]. This protection is further strengthened by exclusive breastfeeding [8]. Children with DS are at increased risk of infections due to qualitative and quantitative abnormalities in both the acquired and innate immune system [9]; hence, the protective qualities of breastmilk are especially beneficial for them [3, 10]. In addition, infants with DS benefit from breastfeeding as it strengthens their tongue‐ and jaw muscles, thus enhancing facial expressionsand language development [3, 10].

Breastfeeding can be a challenge for any mother, but especially having an infant with feeding difficulties. An unsuccessful breastfeeding experience might have a profound impact on the mother and create feelings of failure and inadequacy, which may follow her through life and become a barrier both in the mother–infant relationship and in breastfeeding future siblings [11].

Children with DS are breastfed less frequently compared with other children [10, 12, 13]. Mothers of children with DS express frustration about the child's inability to suck and fear inadequate milk supply [13]. Previous research reveals that mothers believe it is impossible to breastfeed a child with DS [14]. However, research has shown that it is possible for children with DS to breastfeed successfully, despite taking longer to establish [14, 15]. Therefore, to establish partial or exclusive breastfeeding, it is important that mothers of infants with DS receive information that it is possible to breastfeed [3, 13] as well as encouragement and support from healthcare professionals [12, 15]. The information and support required by mothers of infants with DS do not differ much from that given to any other mother. The only difference concerns advice regarding the surrounding environment, such as keeping the light on when breastfeeding or specific breastfeeding positions to provide extra support for the child's head, neck and chin [10].

Previous research shows that despite the benefits, children with DS are breastfed less frequently than other children [10, 12, 13]. More knowledge is needed to increase the frequency of breastfeeding in this vulerable group. Mothers are a source of information, but there is limited knowledge about breastfeeding a child with DS from the perspective of mothers [10, 12, 14, 16, 17], which could be used to develop interventions to support breastfeeding of children with DS.Therefore, the aim of this study was to describe how mothers of children with DS experienced breastfeeding and breastfeeding support from healthcare professionals.

## METHOD

### Study design

A qualitative descriptive method with an inductive approach was used. The data were collected through individual interviews and analysed by inductive content analysis [18, 19].

### Preunderstanding

The authors' experiences of caring for newborns and their parents ranges from 3 to 20 years, and all have met mothers of infants with DS during the neonatal period. Prior to the study and throughout the data collection and analysis, the authors attempted to become aware of their preunderstanding by constantly discussing and reflecting on it in relation to the research process and the phenomenon under study [19]. On occasions when we had differing views, we returned to the interviews and discussed them again to ensure that the result was based on the data and not the authors' preunderstanding.

### Settings and participants

Midwives in Sweden inform mothers about breastfeeding in general at regular maternity care visits. Further information is given to mothers after delivery in combination with highlighting the importance of early skin‐to‐skin contact [4]. The general guidelines are similar irrespective of whether the child is cared for at a maternity ward or neonatal unit. The children of mothers in this study were cared for in a maternity ward, neonatal unit or both.

The mothers were recruited through an advertisement on the Swedish DownSyndrome Association's webpage (www.fub.se) and on a closed Swedish Facebook community called ‘Wonderful Children with Down's Syndrome’. The advertisement was also placed in nine different children's habilitation receptions in the south of Sweden after informed consent was obtained from the manager of each department.

Seven Swedish‐speaking mothers of children with DS who had tried to initiate breastfeeding at the hospital, or later at home, in accordance with the same breastfeeding guidelines given to all new mothers, were included. Their child was under the age of 3 years at the time of the interview in line with the inclusion criterion. The mothers contacted the second author by email or telephone. They received extensiveoral information about the study, after which written information and a consent form were sent to them. When the consent form was returned, the mothers were contacted by phone and a time and place for the interviews arranged.

Five of the mothers chose to be interviewed in their own home, one in a secluded place in a café, and one in a room at the open pre‐school. Prior to the interview, the mothers were once again informed about the study.

The age of the mothers varied between 30 and 45 years, four had a University education, two an upper secondary school education and one an elementary school education. They comprised both first‐time mothers and mothers with previous experience of breastfeeding. At the time of the interview, the children's age varied between <1 and 3 years.

### Data collection

Semi‐structured interviews were conducted by the second and third author. An initial open question was posed: ‘You initiated breastfeeding of your child diagnosed with Mb Down, can you tell me about your experiences and the support you received?’ Supplementary questions for clarification such as: ‘Can you give me an example?’ were posed to give the mothers an opportunity to expand on their experiences. When necessary, the interviewer summarised to ensure she had understood correctly. The interviews lasted 15–44 min (mean = 35) and were audio‐taped and later transcribed verbatim.

### Data analysis

A qualitative content analysis was conducted [18, 19]. A content analysis with an inductive approach was chosen to reveal and describe new knowledge, as the phenomenon under study was considered to be sparsely investigated. In the first step, all authors individually read the transcribed interviews to gain an overall picture of the content and a naive understanding. In the next step, the second and third author identified meaning units (MUs), that is sentences and phrases containing information pertinent to the aim. Subsequently, the authors condensed and coded the MUs. Codes with similar content were merged and then sorted into subcategories and categories based on differences and similarities. All authors discussed the MUs, codes and categories until agreement was reached. The interviews were then re‐read to confirm that all statements relevant to the aim were included in the categories and subcategories. Finally, the authors discussed the content of the interviews and achieved consensus regarding the subcategories and categories. Quotations from the interviews are used to illustrate the result. The letter ‘M’ refers to mother followed by a code number, 1–7.

### Ethical considerations

The study was conducted according to the World Medical Association Helsinki Declaration [20] regarding confidentiality, information about the study, time commitment, and the right to withdraw at any time during the interview or later without giving a reason. All mothers received both oral and written information about the study, and written informed consent was obtained. Regarding confidentiality, the mothers were assured that no personal characteristics would be presented in the results. The study was approved by the Regional Ethics Committee (Dnr. 2015/646).

## RESULTS

The analysis gave the impression that breastfeeding was important to the mothers. However, they did not always receive the support they requested. They experienced that they often had to rely on their ability to find relevant knowledge and guidance, thus requested more person‐centred care. Three categories emerged; *Dealing with the unexpected, Handling preconceptions and Needing information and support*. The subcategories are presented in Table 1.

**TABLE 1:** Categories and subcategories

Dealing with the unexpected	Handling preconceptions	Needing information and support
Needing to find inner strength	Impact of commitment	Depending on person‐centred information
Finding balance in everyday life	Impact of the diagnosis and medical issues	Depending on finding information themselves

### Dealing with the unexpected

The mothers expressed that it was important and meaningful to them to breastfeed their child. They stated that breastfeeding created a feeling of normality in the overwhelming and unexpected situation of becoming a parent to a child with DS. They found an inner strength when their effort to initiate breastfeeding became strenuous.

#### Needing to find inner strength

In general, all the mothers were determined to breastfeed and kept on trying different ways to make it work, celebrating small steps along the way such as removing the feeding‐tube or nipple‐cover or even ceasing to use the pump. When they questioned their own determination regarding breastfeeding, they tried to trust their firm conviction that they would be able to establish it.

…if I had not had the will myself and had not had children before I believe that I would have probably listened more to all the negative things … (M5)

The mothers became stressed as a result of the different strategies surrounding their child's feeding situation, such as complementary feeding and breast pumping. This stress could also impact on their ability to lactate when they used the breast pump, which created a vicious circle of inadequate milk supply and further stress. This resulted in them having to seek strength within themselves to continue trying to establish breastfeeding.

… In early April … it was a period that was really, really hard … then I felt … I cannot get enough milk … when I pumped … I had … a little … in … the freezer … and … saw … how it just became less and less … It was really mentally tough … then … I thought … now … I have … reached the limit … I don't give a damn … (M1)

Eventually, the mothers found their own way of dealing with the difficulties related to breastfeeding their child with DS. This could be that they changed their mind about breastfeeding or just kept on fighting. Some of the mothers described it as a relief when they decided to stop using the breast‐pump and use formula as a supplement when breastfeeding was insufficient. That they had tried was a strength in itself.

#### Finding balance in everyday life

The mothers described that when they received the diagnosis they felt stressed, lonely and concerned about the future. Breastfeeding their child was a way to find balance in life in an overwhelming and unexpected situation. It created normality in a situation that was difficult to grasp. The mothers stated that the knowledge of the positive effects of breastfeeding on mother–child bonding was important for their motivation and helped them to endure when they struggled encountered difficulties.

… I kept on breastfeeding … because I knew … that breast milk is good for them … and that I created a strong bond to him. (M2)

Mothers with previous breastfeeding experience stressed that they did not consider that the child's diagnosis should have any impact regarding the ability to breastfeed. They felt confident in their ability to breastfeed and produce enough breastmilk for the child's needs.

I already have three children and breastfeeding worked out great with all of them … I … did … not … see any … reason … why it shouldn´t work this time … (M3)

Furthermore, they did not accept that their child with DS should be singled out and treated differently to other children. For them, breastfeeding was a kind of statement that their child with DS was as much loved and valued as anyone else.

### Handling preconceptions

The mothers experienced that they were dependent not only on healthcare professionals' commitment but also on attitudes regarding breastfeeding a child with DS. The healthcare professionals' commitment and attitudes could facilitate but also create obstacles.

#### Impact of commitment

The mothers felt that some healthcare professionals had negative attitudes about breastfeeding a child with DS, which could lead to them informing the mother at an early stage that most children with DS could not breastfeed. In cases where the mothers felt that the healthcare professionals had decided that they could not breastfeed their child, they felt neglected as a mother. They expressed that even when they sensed that the healthcare professionals wanted to help them, their belief that it would not work shone through and their attempt to help failed.

… they never said … it is no use … but … it was the feeling you got … (M6)

The mothers experienced that healthcare professionals did not place much emphasis on or talk about breastfeeding. They never asked about the child's feeding pattern or how the mothers experienced breastfeeding their child. The mothers stated that the staff generally focused more on the child's weight and entering the figures into computer charts.

… at the child care center … we almost never talked about breastfeeding … I do not think they prioritized it … (M2)

Those mothers who succeeded in establishing breastfeeding without major difficulties stated that healthcare professionals were positively surprised and sometimes impressed that they could breastfeed their child with DS, which made the mothers feel proud of their accomplishment. At the same time, the mothers felt that the reaction was a bit exaggerated because they considered breastfeeding as something completely normal.

… I wondered why they were so surprised all the time … it was a little excessive in some way… (M7)

The mothers also related that healthcare professionals could be supportive and respond to their wish to breastfeed their child. On such occasions, the mothers did not have to put it into words themselves, as it was somehow understood and unquestioned by the healthcare professionals.

#### Impact of the diagnosis and medical issues

The mothers felt that healthcare professionals were sometimes bound to internal breastfeeding guidelines and routines that were discordant with the families' wishes and the child's needs and ability. The mothers also experienced that the diagnosis itself and associated complications were focused on and breastfeeding received less attention. The healthcare professionals tended to place more emphasis on the syndrome than initiating breastfeeding. The mothers were grateful that healthcare professionals took care of their child's medical needs but still desired assistance when breastfeeding and disliked being left alone due to the fact that healthcare professionals had other priorities.

… In the NICU … there was not much focus on breastfeeding … because there was so much else… (M2)

From the mothers' perspective, it was easier to slip into general routines given to them by healthcare professionals than to ask for advice based on their child's needs. They sometimes did not understand the breastfeeding advice they received, and as there was no follow‐up, they found it difficult to grasp it. This left the mothers alone with a sense of being torn between following their gut instinct regarding breastfeeding or the recommendations given.

I wanted to … remove … the nipple cover … the district nurse said … do not rush … what … did she mean by that … not to rush … try … the second day … or … after two weeks … After about a month … it [the breastfeeding] didn't really work out … then I just removed it [the nipple cover], and let him try … when suddenly, he sucked directly on the breast … (M1)

The healthcare professionals focused more on the feeding situation in its entirety and breast pumping according to the guidelines and routines instead of supporting the initiation of breastfeeding based on the child's need. This sometimes resulted in healthcare professionals suggesting bottle‐feeding to give the child an opportunity to ‘learn’ the sucking technique to remove the feeding‐tube. At the same time, the mothers felt that the healthcare professionals emphasised breastmilk as valuable for the child and motivated them to use the breast pump to obtain a sufficient amount of breastmilk. The mothers experienced this as positive, but would have preferred more individualised breastfeeding advice.

### Needing information and support

The mothers stressed that they wanted breastfeeding support based on their individualised needs and described that they instead received more general standard information that did not meet their individual needs. When the mothers felt that the information was not based on their needs, they had to depend on themselves to find the required information. Furthermore, mothers expressed that they only received support when they asked for it.

#### Depending on person‐centred information

The mothers felt that they were not given any person‐centred information regarding breastfeeding in general or what was involved in breastfeeding a child with DS. They lacked information about the positive effects of breastfeeding a child with DS, such as improved tongue motor skills, language development and immune system. The mothers deemed this type of information important, as they considered such benefits valuable for their child's future. However, the mothers also stated that healthcare professionals provided a great deal of other information regarding the DS diagnosis, which in itself was positive.

You should not take it for granted that it is impossible to breastfeed a child just because it has Down's syndrome. (M7)

#### Depending on finding information themselves

The mothers felt that they had to play an active role in searching for information themselves. Searching for information through the internet and the Swedish Nursing Mothers' Support Group was a way for the mothers to enhance their knowledge. They searched for information about different breastfeeding techniques, tools and information about breastfeeding and DS in general to learn more about how they could help their child.

… later … I searched information by myself online … like how to phase out the nipple cover … (M4)

The mothers also found support and information through peer support groups on Facebook, including peer support groups forfamilies with children who have DS. They also searched for blogs written by other mothers who had been in a similar situation. Peer support groups and blogs were a way for the mothers obtain support and help from others with similar experiences. They expressed that the mothers they met through peer support groups or blogs empowered them in their breastfeeding efforts and gave them a feeling of not being alone, which strengthened them as they had someone to relate to.

… Googled … found … a blog, a woman who also had a premature baby and had started breastfeeding later … it … motivated me a lot … it was important … that type of information… (M1)

The mothers also described that in some cases, they shared what they had learned through the internet with the healthcare professionals to give them information that could be of useful for other mothers. They were surprised when they realised that healthcare professionals already had this knowledge but had not shared it with them. The mothers stressed that it would have been valuable to have received this information from the healthcare professionals, who could have shown them what to do instead of just reading about it on the internet and having to figure it out for themselves.

## DISCUSSION

The results of our study provide insights into mothers' experiences of breastfeeding a child with DS and the support given by healthcare professionals. The mothers had a desire to breastfeed as in some way it normalised their unexpected situation. However, the support they received from healthcare professionals did not always promote breastfeeding. When the mothers lacked information and support, they found their own way of obtaining it, for example the Internet.

The mothers in this study described a desire to breastfeed and seemed aware of the positive effects of breastfeeding a child with DS, such as reinforcing the child's immune system and stimulating the oral muscles [9]. The mothers stated that the healthcare professionals focused on the child's diagnosis and placed more emphasis on the problems that could accompany the diagnosis than supporting the initiation of breastfeeding. This is also confirmed in studies by Sooben [17, 21] and Neila‐Vilen et al. [22], who showed that for healthcare professionals breastfeeding is secondary to the child's medical condition.

Exclusive breastfeeding is recommended in Sweden during the child's first 6 months and according to the WHO [5], breastfeeding offers health benefits for a child, promotes the bonding process and is seen as a natural way to nurture a child. Promoting the bonding process between mother and child is highly relevant and important when the child is born with a lifelong condition such as DS, as the diagnosis might result in anger, shock and sorrow [17]. Bonding can also be promoted by ensuring closeness between the mother and child. Zero separation [23] and skin‐to‐skin contact [24], both of which were of importance for the mothers in this study, are care interventions that promote closeness, support the development of the parental role [25, 26] and enhance breastfeeding [27].

It was evident in our study that mothers requested support based on the child's and their own individual needs. As reported by Hansen [28] and Sooben [17], the support of healthcare professionals is of importance as the care provided can be either an inhibiting factor or a facilitator in the lactation process. For the mothers in this study, it was clear that some healthcare professionals had preconceptions regarding breastfeeding a child with DS. When providing breastfeeding support, the attitudes of healthcare professionals are considered just as important as their breastfeeding knowledge [22, 29]. As described by Hansen [28], if healthcare professionals have an attitude that it is not possible to breastfeed a child with DS, it may act as an incentive for the mothers to reject breastfeeding. It is therefore important that healthcare professionals are aware of their attitudes towards breastfeeding a child with DS, as mothers often see through them. As reported in a study by Ekström and Thorstensson [29] investigating mothers' experiences of healthcare professionals' breastfeeding attitudes, positive attitudes among healthcare professionals lead to mothers being more satisfied with the support.

The study revealed that the mothers implicitly asked for more person‐centred care based on their own and the child's individual needs and ability. This implies a flexibility in guidance and support instead of strictly following guidelines and routines. If healthcare professionals are to support mothers' intention to breastfeed, they need to gain an understanding of each mother's own perspective and emotional experiences to provide more person‐centred support [30, 31]. There is no uniform definition of the concept of person‐centred care, but according to the Centre for Person‐Centred Care in Sweden [32], an important component is the person's own story and a partnership between the patient/relative and the healthcare provider, which is also in line with the Institute for Patient‐ and Family‐Centered Care [33]. In person‐centred care, person‐centred communication based on individualised and empathetic listening [34, 35] is crucial. Respectful communication between mothers and healthcare professionals can lead to mutual understanding, resulting in significant support for the mothers. Person‐centred care with caring communication can meet the needs of those mothers who are distressed in their quest to either continue trying to establish breastfeeding or accept the introduction of formula as a supplement to breastfeeding.

Lack of support from healthcare professionals resulted in the mothers in this study searching for information from other sources such as the Internet and peer‐support groups. This way of seeking information is similar to previous studies, which also shows that peer support via social media can be a good way to gain advice and information. Peer‐support groups on the internet can be an important information resource for breastfeeding mothers [36]. Mothers regard closed peer support groups as a community where they can meet other breastfeeding mothers with similar experiences who can give them support and information when they need it. The communities also give the mothers confidence and empower them in their parenting [37].

In earlier studies, breastfeeding support from a partner, other next of kin or some sort of ‘safety net’ was shown to be positive [38, 39] in a situation where both parents are affected by their child's condition. Having a child with DS affects both parents, but in our study, the mothers did not mention any support from the fathers or other next of kin. This can of course be due to many causes. However, if healthcare professionals use a person‐centred approach [30], they could obtain valuable knowledge about what support the mother needs, which might be a gateway to more actively involving the other parent.

The strength of our study is that the children's ages range from <1 to 3 years, while the mothers represent both first‐time mothers and mothers with previous experience of breastfeeding. Despite this, the mothers have similar experiences about their breastfeeding situation, which indicates that there has been no change overtime regarding support for mothers who give birth to a child with DS. Another strength is that the mothers were recruited from different regions in southern Sweden, which might minimise the effect of local caregiving routines. The analysis process has been described as precisely as possible to increase credibility [40]. During the analysis process, the researchers had an open dialogue where they compared and reflect on the data. By using quotations from the mothers, the objectivity of data has been strengthened [18].

A limitation of this study is the small sample size. One reason is that only 1.5/1000 children are born with DS in Sweden [2], which makes it difficult to find mothers with experience of breastfeeding a child with DS. Despite the fact that we searched for mothers through three different children's habilitation receptions, a closed Swedish Facebook community and the Swedish Down Syndrome Association's Webpage we were only able to recruit seven mothers. However, according to Malterud, Siersma and Guassora [41], a small sample can be acceptable in terms of information power when the aim is narrow, the research question more focused and the participants are ‘experts’ on the phenomenon under study, thus contributing rich and relevant descriptions. Another limitation is that all the included mothers were of Swedish origin. It is important to bear in mind that mothers with another ethnicity might experience the phenomenon differently.

## CONCLUSION AND IMPLICATIONS

Mothers are sensitive to the attitudes of healthcare professionals and feel that negative attitudes can affect their own state of mind and how they experience the support provided. The mothers felt that healthcare professionals were bound to ward routines and guidelines, which could conflict with their own wishes, as well as the child's needs and ability. Mothers of children with DS need person‐centred support and healthcare professionals require specialist knowledge to provide such support. One way to support these mothers might be to establish a team including healthcare professionals from the maternity ward and neonatal unit who have in‐depth knowledge about breastfeeding children with special needs such as DS. Preconceptions regarding breastfeeding and DS have not changed over the years, even though research shows that infants with DS are able to breastfeed successfully. There is a need for further research within this field to support mother–infant attachment and the mother's wishes regarding breastfeeding. It would be of great value if future research focused on designing and implementing support to increase knowledge regarding breastfeeding and DS among healthcare professionals.

## AUTHOR CONTRIBUTIONS

Lisbeth Jönsson (LJ) planned the study together with Christina Olsson Tyby (COT) and Sara Hullfors (SH), participated in the data analysis and writing and reviewing of the manuscript. COT and SH conducted the interviews, and participated in the data analysis and writing the manuscript. Pia Lundqvist participated in planning parts of the study, contributed by assisting with the data analysis, writing, and reviewing of the manuscript. All authors read and approved the final manuscript.

## CONFLICT OF INTERESTS

There is no conflict of interest to report.

## ETHICAL APPROVAL

The study was approved by the Regional Ethics Committee (Dnr. 2015/646).
